# A systems biology analysis of protein-protein interaction of digestive disorders and Covid-19 virus based on comprehensive gene information 

**Published:** 2022

**Authors:** Arghavan Hosseinpouri, Mostafa Rezaei-Tavirani, Elham Gholizadeh, Reza Karbalaei

**Affiliations:** 1 *Department of Cellular and Molecular Sciences, Faculty of Sciences, Persian Gulf University, Bushehr, Iran*; 2 *Proteomics Research Center, Faculty of Paramedical Sciences, Shahid Beheshti University of Medical Sciences, Tehran, Iran*; 3 *Department of Biochemistry, Faculty of Medicine, Semnan University of Medical Sciences, Semnan, Iran*

**Keywords:** Digestive system disorders, Covid-19, IBD, Systems biology, Diarrhea, Gastritis

## Abstract

**Aim:**

Analysis of networks of digestive disorder and their relationship with Covid-19 based on systems biology methods, evaluation similarity, and usefulness of networks to give a new treatment approach.

**Background:**

Digestive disorders are typically complex diseases associated with high treatment costs. They are related to the immune system and inflammation. With the outbreak of Covid-19, this disease was shown to have signs like diarrhea. Some signs of Covid-19 are similar to those of digestive disorders, like IBD and diarrhea. Both of them are accompanied by inflammation and induce disorders in the digestive system.

**Methods:**

DisGeNET and STRING databases were sources of disease genes and constructing networks and were used to construct the network of digestive diseases and Covid-19. Three plugins of Cytoscape software, namely ClusterONE, ClueGO, and CluePedia, were used to analyze cluster networks and enrichment pathways. To describe the interaction of proteins, information from KEGG pathway and Reactome was used.

**Results:**

According to the results, IBD, gastritis, and diarrhea have common pathways. The CXCL8, IL-6, IL-1β, TNF-α, TLR4, and MBL2 molecules were identified as inflammatory molecules in all networks.

**Conclusion:**

It seems that detecting genes and pathways can be useful in applying new approaches for treating these diseases.

## Introduction

 Coronavirus from the large Coronaviridae family can cause diseases in humans and birds. SARS-CoV2 (Covid-19) was detected for the first time in 2019 in Wuhan, China, and three months later, it was widespread in the world and led to a pandemic (1). To date, physicians have reported many signs and symptoms of Covid-19, such as its effects on the digestive tract, nervous system, blood circulation, heart, lung, and kidney (2, 3). Studies have also reported that the coronavirus implicates 1844 genes, some of which are common among other diseases. The genes involved in digestive disorders like cholysititis, diarrhea, gallstone, gastritis, irritable bowel disease, stricture, and acid reflux also are common for Covid-19 (4).

Cholelithiasis is inflammation of the gallbladder; this disease is not well understood, but some similarities between cholelithiasis and gall stone disease have been discovered (5). Diarrhea is a disease defined by abnormal fluidity in stools, and this is common in developing countries, specifically Africa, shown by the high mortality rate in children (6). A recent study done by Li et al. (2020) described diarrhea as the most common symptom (fifth main symptom) in Covid-19 patients (7). Gastritis is known as the acute or chronic inflammation of the stomach lining (8). There are many reasons for gastritis, such as alcohol abuse and the long-term intake of non-steroidal anti-inflammatory drugs (9). Recently, studies have shown patients with gastritis and inflammatory bowel disease (IBD) are at high risk of Covid-19 because of the high activity of ACE2 in their plasma (10-12). Sometimes Covid-19 can weaken patients with IBD, and it may lead to their death (13, 14).

**Table 1:** The enrichment of three modules for diseases

**Disease**	**Adjusted p-Value**	**Module**
**Immunodeficiency**	**4.070772946660061E-4**	**Blue module**
**Macular Degeneration **	**2.447422407186003E-6**	**Green module**
**Anemia**	**5.473352213598934E-9**	**Red module**

A stricture occurs in intestinal fibrosis and collagen accumulation (15). The main reasons for stricture are cancer and inflammatory bowel disease; Crohn’s disease is also a cause of stricture in the small bowel (16). Based on recent findings, there are some common genes between stricture and Covid-19 (4). Gastroesophageal reflux is a common digestive disorder that affects millions of people worldwide (17). In addition to risk factors like diet, smoking, and body mass index, it has been suggested that some genes are important for acid reflux (18, 19). This study aims to investigate common genes and protein-protein interactions among the mentioned diseases and Covid-19 infection to find the similarities between gene pathways. It seems that finding the same gene pathway in those diseases can help find an approach to treating Covid-19 infection. 

## Methods

DisGeNET is an available platform that includes one of the largest collections of genes and variants associated with human disease (20). The related genes of digestive disorders (mentioned in the introduction) and Covid-19 were exported from the DisGeNET database and used to construct the PPI network. The Search Tools for Detecting Interacting Genes/Proteins (STRING), a database for predicted protein-protein interactions at EMBL, clusters the elicited results from many protein-protein interactions databases, like Mint, BioGrid, etc. To describe the interaction of proteins, information from the KEGG pathway at the address www.genome.jp/kegg/ and Reactome at address www.reactome.org was used (21). We constructed digestive disorders and Covid-19 networks by submitting the list of genes to the STRING database at the address www.string-db.org and analyzed the networks by Cytoscape software at the address www.cytoscape.org (22). 

A network includes nodes (e.g., genes or proteins) and links/edges (e.g., co-expression relationships or physical interactions). In biology, network terms, degree, and betweenness are central parameters for analyzing network topology. Edges/links of a node are named the degree of that node. Nodes with high degrees are called hubs, and nodes that achieve top-ten or top-five percent of betweenness centrality are called bottlenecks (both based on the researcher’s definition) (23). Therefore, the nodes that simultaneously have hubs and bottlenecks are called hub-bottlenecks (24). The standard deviation (SD) and average degree (AD) were calculated, and nodes with more than two *SD + AD were selected as hub proteins in each network. In addition, the top five percent of betweenness centrality measures were selected as bottleneck proteins. Common genes, hubs, and bottleneck proteins of these Covid-19 and digestive disorder gene networks were extracted and used for more analysis. The common networks were constructed by importing common genes in the STRING database and clustered by the ClusterONE plugin of Cytoscape software (25). This software found overlapping protein complexes in a protein interaction network uploaded into Cytoscape (overlap threshold = 1, node penalty = 0, haircut threshold = 0) (26). By ClueGO and CluePedia plugins of Cytoscape software, pathway enrichment and the relation between pathways were accomplished (27, 28) (table 1). 

## Results

**Figure 1:**
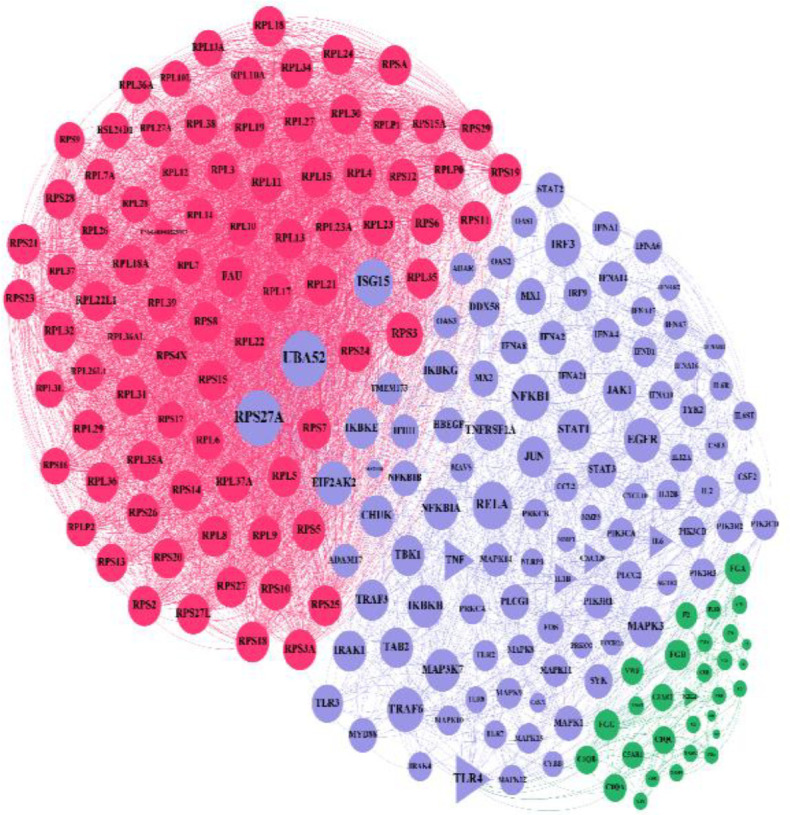
Common gene network containing 219 nodes and 4932 Edges. This network includes three modules that are shown by blue (cluster one), red (cluster two) and dark green (cluster three)

Using the DisGeNET database, we extracted 844 genes for Covid-19, 56 genes for cholecystitis, 631 genes for diarrhea, 52 genes for gallstone, 293 genes for gastritis, 428 genes for irritable bowel disease (IBD), 29 genes for stricture, and 51 genes for acid reflux; 219 genes were shared among all diseases. Three diseases, i.e. IBD, gastritis, and diarrhea, showed some common genes with Covid-19, but the other three diseases showed no common gene. Using the STRING database, the common genes network was constructed. The Covid-19 network showed 219 nodes and 4932 edges (Figure 1). 

The nodes have been colored in green; Covid-19 genes and some of those were colored red, because they were common with IBD, gastritis, and diarrhea genes.

**Figure 2:**
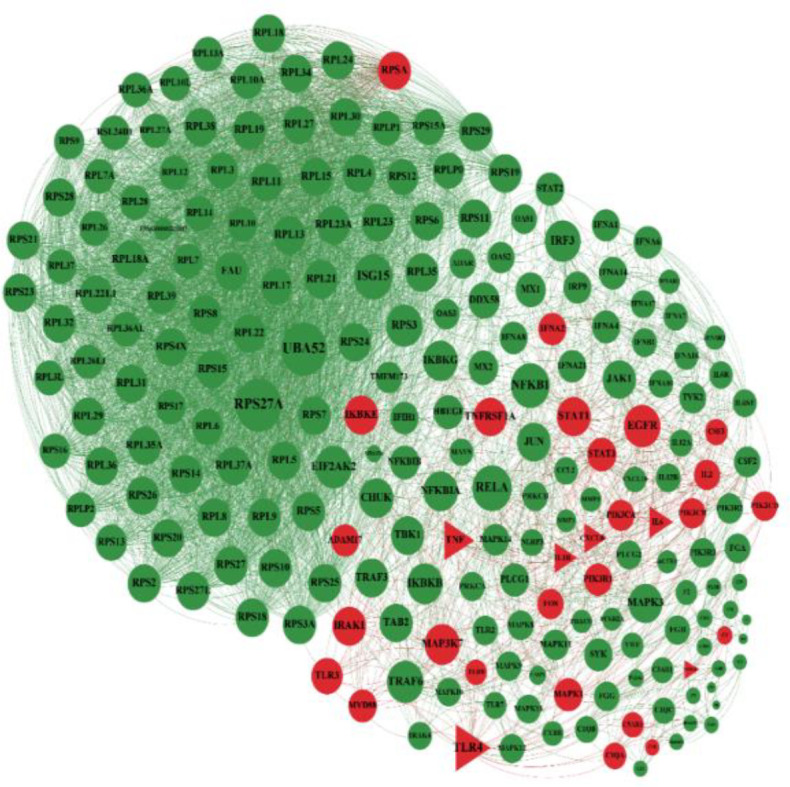
Common genes between Covid19 and diarrhea. Involved genes in covid19 are shown with green and common genes with diarrhea are red

**Figure 3:**
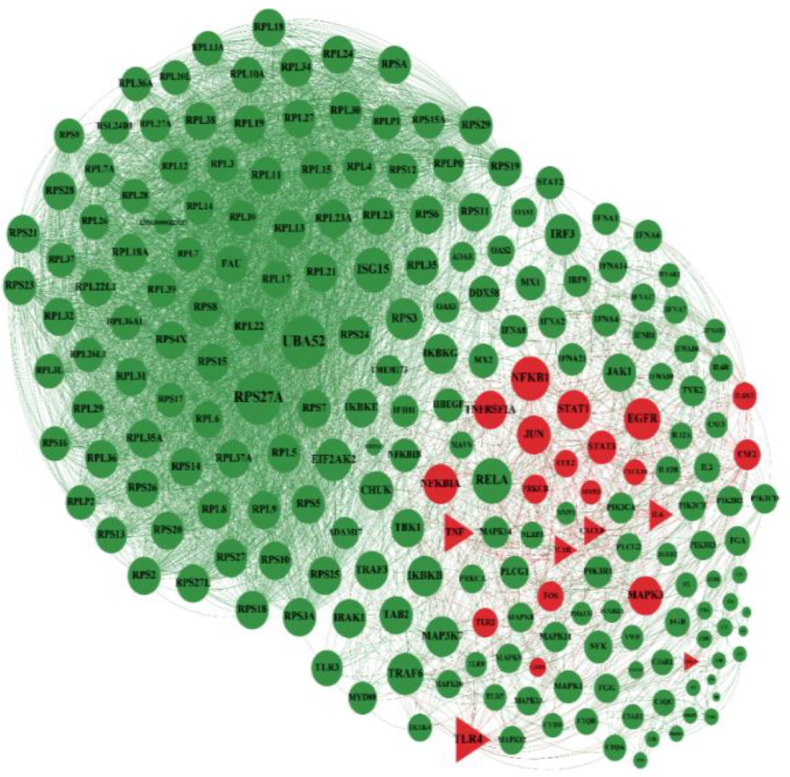
Common genes between Covid19 and gastritis. The genes were colored by green are involved in covid19 and common genes with gastritis are in red

**Figure 4:**
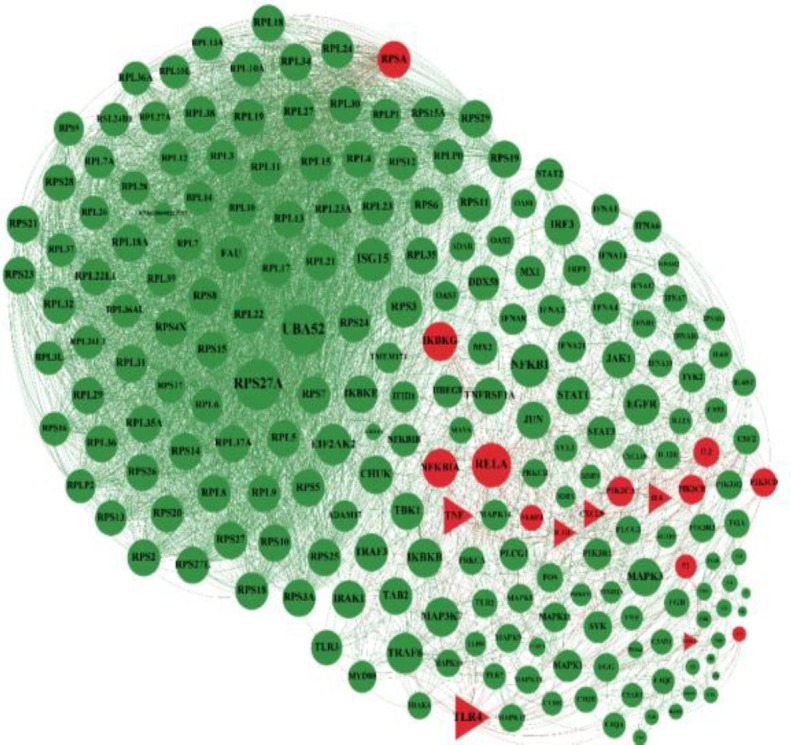
Common genes between Covid19 and IBD. The genes in green color are involved in covid19 and common genes with IBD are red

In the next step, the general network of Covid-19 genes was illustrated in which common genes with diarrhea are shown in red (Figure 2). The network of Covid-19 genes showed that some of these genes were common with gastritis. The nodes colored green are Covid-19 genes, and common genes with gastritis are colored in red (Figure 3). The association of Covid-19 genes and IBD has been shown. The nodes in green color are Covid-19 genes, and common genes with IBD are in red (Figure 4). The genes that are common between the three diseases are shown by a triangular shape.

## Discussion

In systems biology science, PPI network analysis and pathway enrichment have been broadly used for discovering main proteins and pathways underlying complex diseases (29). Different types of disorders such as neurodegenerative and many cellular conditions have been analyzed (30, 31, 32, 33, 34, 35). 

In this study, we obtained the gene list of six diseases (mentioned above) that seemed to have a common molecular mechanism. Among those, three diseases (IBD, gastritis, diarrhea) had a common mechanism with Covid-19 based on risk factors and studies (36, 37, 38, 39). 

CXCL8 (IL-8) molecule is a member of the chemokine family that causes inflammation and pathogen elimination. Based on the PPI network, six genes, comprising CXCL8, IL-6, IL-1β, TNF-α, TLR4, and MBL2, were common in the inflammation network. Many studies have suggested that CXCL8 may also have a role in tissue injury, fibrosis, angiogenesis, and tumorigenesis. Thus, many cells like epithelial cells, fibroblasts, and neurons express CXCR1 and CXCR2 as receptors for CXCL8, and CXCL8 can cause neutrophil and macrophage migration. In inflammation diseases, they, mostly neutrophils, function as attractive chemokines to leukocyte migration. Studies have shown that the blockade of CXCL8 can prevent inflammatory diseases (40). In addition, TNF-α, IL-1β, IL-6, vascular endothelial growth factor (VEGF), and CXCL8 can play important roles in inflammatory diseases such as osteoarthritis, IBD, and infections like Covid-19 (41).  

Wojdasiewicz et al. (2014) described the inflammatory potential of IL-1β, TNFα, IL-6, IL-15, IL-17, and IL-18, stating that they disturb tissue homeostasis and then, with the attraction of leukocyte cells, induce inflammation (42). 

According to recent studies, the digestive disorders caused by Covid-19 have inflammatory signs and the same genes involved in digestive diseases that patients suffer from immune deficiency and mutation disorders. 

The Sars-cov2 virus usually fuses to cells by the ACE2 receptor. Typically, the expression rate of this receptor in the digestive system is high, so cells in this region are reservoirs for the Sars-cov2 virus and cause inflammation and stimulation of immune responses. In stimulating an immune response, IL-1β, both independently and combined with other inflammatory cytokines, can affect the expression of adhesion molecules and induce signals to activate inflammatory molecules such as TLRs. Therefore, IL-1β can act as a helper to activate immune responses (43). 

In addition, many studies have described the function of TNF-α like IL-1, and both of them affect the hepatic cells to induce the expression of complement peptides such as MBL2 (44). 

TNF-α and IL-1 affect respiratory cells and thus decrease the efficiency of respiration. TNF-α, IL-1, and IL-6 also affect the hypothalamus, induce fever, and stimulate an immune response (45, 46). TNF-α is responsible for the expression of IL-6, IL-8 (CXCL8), RANTES (CCL5) and VEGF (47, 48). The study results showed that TNF-α, IL-1β, IL-6, TLR4, and MBL2 are common among Covid-19, IBD, gastritis, and diarrhea. These molecules induce inflammation and an immune response (49, 50, 51, 52). 

It seems that methods used to treat diarrhea, IBD, and gastritis can be effective in treating Covid-19, according to their similar pathway and gene involvement. Moreover, identifying similarities in pathways and genes can give us a new approach to applying genes for therapy and for use as markers for diagnoses, albeit more studies are needed.

## Conflict of interests

The authors declare that they have no conflict of interest.
